# Supplementary material for: A Critical View of the Application of the APEX Software (Aqueous Photochemistry of Environmentally-Occurring Xenobiotics) to Predict Photoreaction Kinetics in Surface Freshwaters
Source: Molecules. 2019 Dec 18;25(1):9. doi: 10.3390/molecules25010009 (PMC7017383; doi:10.3390/molecules25010009)
Supplement: Supplementary file 1 [file molecules-25-00009-s001.zip › 3.2.4_gcc-4.4.0.zip!/3.2.4_gcc-4.4.0/3.2.4_gcc-4.4.0/doc/octave/html/interpreter/Statistics.html]

Statistics - Untitled


Next: Sets,
Previous: Optimization,
Up: Top


---

## 25 Statistics

Octave has support for various statistical methods. This includes
basic descriptive statistics, statistical tests, random number generation,
and much more.

The functions that analyze data all assume that multidimensional data
is arranged in a matrix where each row is an observation, and each
column is a variable. So, the matrix defined by

```
     a = [ 0.9, 0.7;
           0.1, 0.1;
           0.5, 0.4 ];
```

contains three observations from a two-dimensional distribution.
While this is the default data arrangement, most functions support
different arrangements.

It should be noted that the statistics functions don't test for data
containing NaN, NA, or Inf. Such values need to be handled explicitly.

- Descriptive Statistics- Basic Statistical Functions- Statistical Plots- Tests- Models- Distributions- Random Number Generation
